# Mild acidic charcoal: adsorption, analysis, and application

**DOI:** 10.55730/1300-0527.3692

**Published:** 2024-08-01

**Authors:** Rajendra PATIL, Jagdish CHAVAN, Shivnath PATEL, Anil BELDAR

**Affiliations:** Department of Chemistry, P.S.G.V.P. Mandal’s SIP Arts, GBP Science, and STKV Sangh Commerce College, Shahada, Nandurbar, India

**Keywords:** Acidic charcoal, xanthenes, adsorption

## Abstract

The adsorption of glacial acetic acid over a charcoal support was investigated. The amount of adsorption was analyzed using a traditional titration method and the prepared adsorbed system was employed as a heterogeneous catalyst for organic reactions as a viable application. Different 14-aryl-14*H*-dibenzo[a,j]xanthenes were synthesized using mild acidic charcoal as a catalyst and yields of 88%–94% were obtained. The advantages of this method include the easy preparation of a cheaper and environmentally safe catalyst system, a simple work-up procedure, and excellent catalytic efficacy.

## 1. Introduction

In the last few decades, heterogeneous catalysis has attracted the attention of chemists due to the benefits of convenient recovery of heterogeneous catalysts from reaction media and efficient recycling for relatively high numbers of cycles. Among the various heterogeneous supports for catalysts, polymeric carbon in different morphological forms has become popular due to special characteristics including a large surface area, chemical inertness, thermal and mechanical stability, and structural uniqueness. Carbon materials including charcoal, graphene, graphene oxide, reduced graphene oxide, and carbon nanomaterials of various nanosized forms were reported as catalysts as well as support for catalysts for many organic transformations [1]. Nanocarbon materials are used for a wide variety of nanoparticles that can be distinguished based on morphology, size, and nature, such as nanotubes (single and multiwall), nanofibers, fullerenes, nanospheres, hollow spheres [2], nanocoils, nanodiamonds, nanohorns, nanoonions, nanocages, nanoleaves, quantum dots, sheet-like carbon or graphene, nanohybrids, and nanoporous carbon [3,4].

Extensive reports about the application of palladium on charcoal in organic synthesis specifically for coupling reactions, oxidation, dehydrogenation, carbonylation, polymerization, cyclization, or hydrogenolysis are available in the literature [5]. Carbon nanoparticles have also been employed as photocatalysts, acid-base catalysts, and electrocatalysts [6]. Nanoparticles incorporating noble metals such as silver (Ag), gold (Au), ruthenium (Ru), and palladium (Pd) have been supported on nanocarbon materials such as carbon nanotubes, and graphene/graphene oxide has been widely employed in promoters and catalysts in many organic transformations [7]. Graphene oxide, reduced graphene oxide, functionalized graphene oxide, and heteroatom-doped graphene have also been employed as metal-free heterogeneous catalysts [8]. Applications of metal oxide nanoparticles supported on graphene/reduced graphene oxide, pyrene-tagged palladium and ruthenium complexes immobilized on reduced graphene oxide, sulfonated reduced graphene oxide, N-heterocyclic carbene metal complexes supported on graphene oxide, and ionic liquid supported on graphene oxide in nanocomposites were reported to yield efficient heterogeneous nanocatalysts for various organic transformations [9,10]. Graphene-based nanocomposites including polymeric carbon nitride nanocomposites (graphene sheet), palladium on graphene, palladium on partially reduced graphene nanosheets, graphene oxide on ferroferric oxide, sulfated graphene, thiolated graphene oxide, silver-decorated graphene oxide catalysts, and manganese oxide nanorods/graphene oxide composites have been employed as nanocatalysts for a variety of organic reactions [11]. In the present work, using a 1:1 (v/w) ratio of acetic acid and activated charcoal, an adsorption catalyst was prepared. The loading of acetic acid on charcoal was determined with random samples of 0.1 g using a titration method against 0.01 N NaOH.

## 2. An overview of charcoal-supported catalysis

A number of polymers have been found to be applicable for heterogeneous solid support, but charcoal has particularly caught the attention of researchers in the field of heterocyclic synthesis because charcoal is a good adsorbent with large surface area, thermochemically stable, greener, cheaper, readily available, and reusable. A variety of charcoal-supported heterogeneous catalysts have been used in different organic transformations, including MoO_2_-Bu_3_SnCl supported on charcoal [12], tungstic acid-tributyltin chloride immobilized on charcoal [13], sulfonated charcoal [14–16], nickel on charcoal [17], polymeric carbon in the form of expandable graphite,[18] palladium on charcoal [19–21], tungstophosphoric acid on activated carbon [22], sulfonated carbon materials [23], H_2_SO_4_-charcoal [24], ZnO- and Nb_2_O_5_ -activated charcoal [25], Pd-Cu on charcoal [26], CuNPs on activated carbon^1^ [27], and NaHSO_4_·H_2_O on activated charcoal [28].

Considering the advantages of charcoal support applications in organic synthesis [29] and transformations, the present study was undertaken to develop a charcoal-supported acid catalyst system for the synthesis of 14-aryl-14*H*-dibenzo[a,j]xanthenes.

## 3. Experimental

All starting reagents were purchased from Loba Chemie Pvt. Ltd. (Mumbai, India) or Merck Specialities Pvt. Ltd. (Mumbai, India) and used without further purification unless noted. Melting points were determined using the conventional method and verified. Reactions were monitored by thin-layer chromatography on silica gel 60 F_254_ plates. Since all obtained products have been reported, they were characterized by melting points with comparisons to those reported in the literature.

### 3.1. Preparation of mild acidic charcoal

In the literature, metal nanoparticles, metal oxide, sulfuric acid, and other materials were reported to be supported on charcoal. To ameliorate the strong and hazardous acid-catalyzed reaction conditions, we aimed to develop a mild acid catalyst system using acetic acid supported on charcoal. To ensure a simple work-up procedure, ease of catalyst handling, reduced catalyst amounts, and the recycling of the cheap, safe solid support, charcoal was chosen for the heterogeneous reaction environment. Different charcoal particle sizes were used, such as granular (1.5 mm) and fine (1 mm). The acetic acid and activated charcoal were combined at a ratio of 1:1 (v/w) (Figure). After the preparation of the acetic acid adsorbed on the charcoal system (using the solvent method described in Section 3.1.2), the loading of the acetic acid over granular and fine charcoal was estimated titrimetrically (using the method described in Section 3.2). Three random acidic charcoal samples of 0.1 g for both fine (samples F1, F2, and F3) and granular (samples G1, G2, and G3) charcoal were used for the estimation of loading. Acidic charcoal samples F1, F2, and F3 achieved acetic acid loading of 14.2, 14.6, and 15 mmol/g (85%–91%), respectively, while samples G1, G2, and G3 respectively achieved loading of 10.9, 11.3, and 11.9 mmol/g (65.4%–71.4%). Thus, the fine charcoal samples had higher amounts of adsorbed acetic acid compared to the granular samples. This could be attributed to the small particle size of fine charcoal, which possesses a large surface area for adsorption. Therefore, fine charcoal was utilized as a support for the preparation of the mild acidic charcoal catalyst.

#### 3.1.1. Neat method

We first undertook the preparation of acidic charcoal under neat conditions [24]. Activated fine charcoal (1 g) was placed in a 50-mL beaker and 1 mL of glacial acetic acid was subsequently added. The mixture was stirred thoroughly with a glass rod, yielding the acidic charcoal catalyst. To estimate the loading of the adsorbed acetic acid catalyst, three random samples (0.1 g) of the previously prepared acidic charcoal were considered (using the method described in subsection 3.2). Surprisingly, the adsorption of the acetic acid on the charcoal was found to be quite uneven, varying from 63.5% to 88.7%. To overcome the uneven adsorption of the acetic acid under neat conditions, we used a solvent [30].

#### 3.1.2. Solvent method

To achieve homogeneous and uniform adsorption of the catalyst over charcoal, ethanol was used as a solvent. Glacial acetic acid (1 mL) was added to a 50-mL round-bottom flask fitted on a magnetic stirrer and containing 10 mL of ethanol. The mixture was then stirred to obtain a homogeneous solution. To that homogenized solution of ethanolic acetic acid, 1 g of activated fine charcoal was added in portions and the mixture was stirred for 10 min for maximum uniform adsorption. The ethanol was then removed with a rotary evaporator to obtain dry acetic acid-adsorbed charcoal powder. Estimation of the adsorbed acetic acid of the three random samples of acidic charcoal prepared by solvent method was performed titrimetrically, as described in the next section. The loading of acetic acid on charcoal was found to be 85%–91%, revealing satisfactory adsorption higher than that obtained by the neat method (Table 1) [30].

### 3.2. Estimation of acetic acid loading on charcoal

The general procedure used for the titrimetric estimation of the amount of acetic acid adsorbed on charcoal involved 0.1 g of acidic charcoal prepared with fine and granular charcoal by neat and solvent methods. Acidic charcoal (0.1 g) was mixed with 10 mL of distilled water in a 50-mL beaker and stirred. The solution was then filtered with a filtration funnel with washing by 5 mL of distilled water in portions. The washing of the charcoal residue with distilled water continued until the eluent did not show any traces of acid as confirmed with blue litmus paper. The collected filtrate was then diluted to 100 mL in a volumetric flask. The standardization of 0.01 N NaOH solution was performed using standard 0.01 N oxalic acid solution. The diluted acid solution (10 mL) was then titrated with the previously standardized 0.01 N NaOH using phenolphthalein as an indicator. The titration procedure was repeated two more times to obtain averaged burette readings. Using the average burette readings and the concentration and volume parameters of both types of solutions, the concentrations of the acid solutions were obtained. Using those calculated concentrations, the molecular weight of the acetic acid, and the volume of the diluted acid solution, the amount of acetic acid adsorbed per 0.1 g of acidic charcoal was determined using the following equation [30]:


W=EW×C(cal)×V

Here, W = amount of acetic acid in g, EW = equivalent weight of acetic acid, C(cal) = calculated normality of acetic acid, and V = volume of the diluted acid solution in liters.

### 3.3. Synthesis of 14-aryl-14H-dibenzo[a,j]xanthenes

The optimization of the amount of mild acidic charcoal catalyst was performed using benzaldehyde and *β*-naphthol (1:2) in refluxing ethanol with different amounts of mild acidic charcoal catalyst (Scheme). The optimized catalytic conditions provided satisfactory results with respect to the reaction time and yield of the compounds at an amount of 0.1 g as described in Table 2. Further increases in the catalyst amount beyond 0.1 g did not cause significant changes in reaction time or yield. Therefore, 0.1 g of mild acidic charcoal was utilized as the catalyst for the validation of catalyst activity over a range of diverse substrates.

A 50-mL round-bottom flask containing a mixture of aldehyde (10 mmol), *β*-naphthol (20 mmol), and 0.1 g of mild acidic charcoal catalyst was refluxed with 10 mL of ethanol for an appropriate time (Table 3). The progress of the reaction was monitored by TLC using an ethyl acetate and *n*-hexane solvent system. After completion of the reaction, the warm reaction mixture was filtered with a filtration funnel to separate the charcoal residue. The charcoal residue was washed with ethanol (3 × 3 mL). The products were obtained from the filtrate after removal of the ethanol by rotary evaporator. The recrystallization of crude products from the aqueous ethanol afforded high yields of the corresponding dibenzoxanthenes.

## 4. Results and discussion

Acetic acid was previously used as the medium for the synthesis of dibenzoxanthenes in the presence of phosphoric acid or perchloric acid as the catalyst [31]. One reported method applied acetic acid and sulfuric acid (4:1) for the preparation of xanthenes [32]. In light of the importance of polymer- or solid-supported catalysis to overcome the use of hazardous and concentrated acids as catalysts, we attempted the preparation and application of mild acidic charcoal as a catalyst. This catalyst system is environmentally friendly and safe to handle. The charcoal support is inert and provides a large surface area for the adsorption of acetic acid together with sufficient surface area for the reactants, which allows the production of high yields. The synthesis of 14-alkyl/aryl-14*H*-dibenzo[a,j]xanthenes was demonstrated using a heterogeneous mild acid charcoal catalyst. Acetic acid and activated charcoal were utilized at a 1:1 (v/w) ratio to prepare the adsorption catalyst, and the loading of acetic acid on charcoal was determined with random samples of 0.1 g using a titration method against 0.01 N NaOH. This revealed 80%–91% acetic acid per 0.1 g of acidic charcoal catalyst. Optimization of the amount of mild acidic charcoal catalyst was performed utilizing different amounts (Table 2) under refluxing ethanol, and 0.1 g of catalyst showed satisfactory results with respect to yield and reaction time. Further increases in the catalyst amount did not produce any remarkable changes in the results. Catalyst efficacy was validated using different substituted aromatic/aliphatic aldehydes and *β*-naphthol in refluxing ethanol (Table 3). Aldehydes bearing electron-releasing and electron-withdrawing groups reacted successfully; the influence of electron-releasing and electron-withdrawing groups was also observed according to the differences in reaction times and yields. These dibenzoxanthenes were already reported in the literature; the melting points of all products were uncorrected with comparisons to those given in the literature.

The efficiency rates of a number of reported catalysts were compared with the proposed mild acidic charcoal catalyst, as seen in Table 4. Some of the catalysts given in Table 4 required long reaction times to achieve maximum catalytic activity compared to the mild acidic charcoal. The product yield provided by acidic charcoal catalysis was found to be comparable.

We also evaluated the reusability of the recovered charcoal support for many cycles after activation and adsorption with acetic acid as per the procedure described for the preparation of the mild acidic charcoal catalyst.

In conclusion, in this study, charcoal of fine and granular nature was utilized with different particle sizes for the preparation of mild acidic charcoal. Experiments showed that fine charcoal adsorbed the maximum amount of acetic acid per 0.1 g. Preparation of a heterogeneous catalyst system was performed using neat and solvent methods, and maximum and uniform adsorption was achieved with the solvent method. The loading of the acetic acid on adsorbed charcoal was determined titrimetrically. The observed results led us to choose fine charcoal and the solvent method for the preparation of mild acidic charcoal, which was then employed as an efficient heterogeneous catalyst for the synthesis of some dibenzoxanthenes. The obtained yields of the desired products were in the range of 88%–94%.

## Figures and Tables

**Figure:**
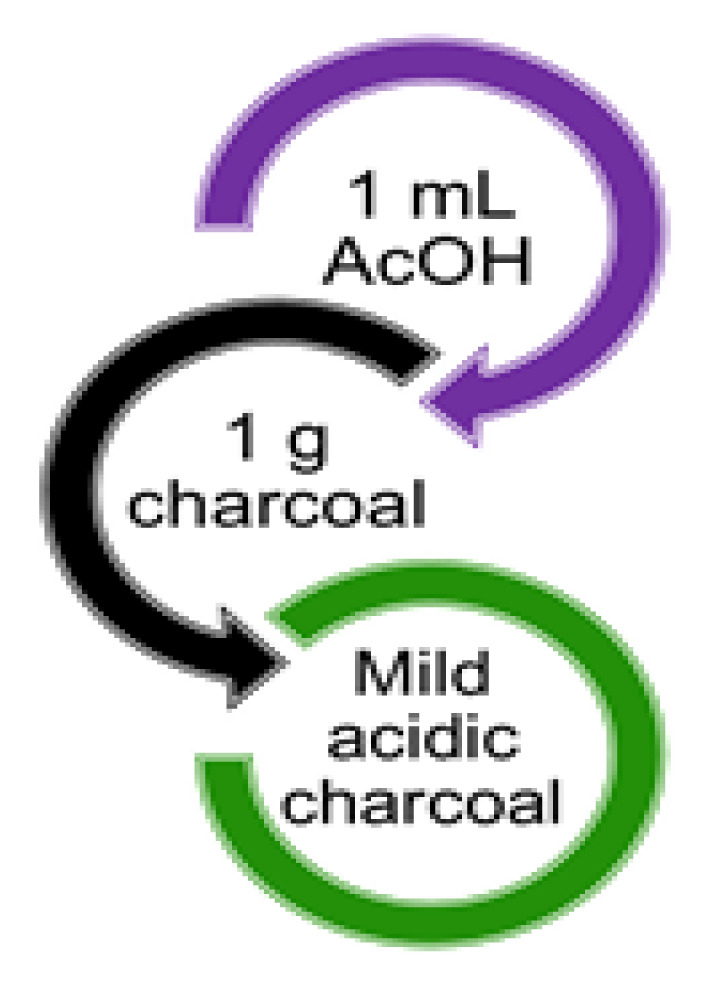
Easy preparation of mild acidic charcoal catalyst.

**Scheme:**
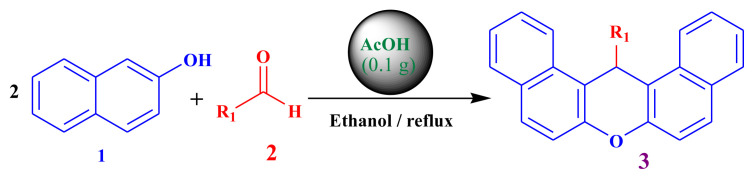
Synthesis of 14-alkyl/aryl-14*H*-dibenzo[a,j]xanthenes.

**Table 1:** Comparative characterization of adsorbed catalysts.

Adsorption method	Solvent Used	0.1 g acidic charcoal Samples	% adsorption
Neat method	-	Sample 1	63.53
		Sample 2	73.69
		Sample 3	88.70
Solvent method	Ethanol	Sample 1	85.31
		Sample 2	91.00
		Sample 3	87.50

**Table 2:** Optimization of acidic charcoal catalyst amount under refluxing ethanol using benzaldehyde and *β*-naphthol (1:2).

Entry	Catalyst (g)	Time (h)	Yield (%)^b^
1	0.025	4	80
2	0.050	4	84
3	0.075	3	88
**4**	**0.100**	**2.5**	**94**
5	0.125	2.5	92
6	0.150	2.5	90

**Table 3:** Synthesis of 14-alkyl/aryl-14*H*-dibenzo[a,j]xanthenes catalyzed using mild acidic charcoal.

Entry	Product	Time (h)	Yield^b^ (%)	Melting point (°C)	
				Observed	Reported
**3-I**	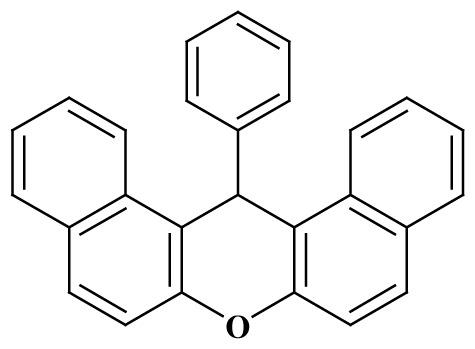	2.5	94	180–182	181–183 [33]
**3-ii**	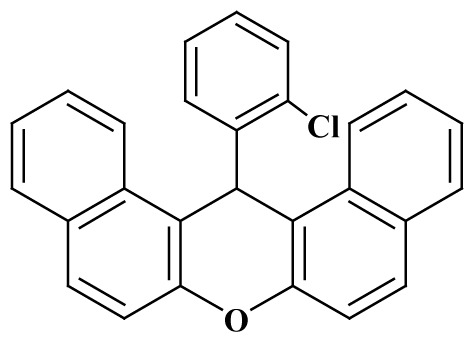	2	90	214–216	214–216 [33]
**3-iii**	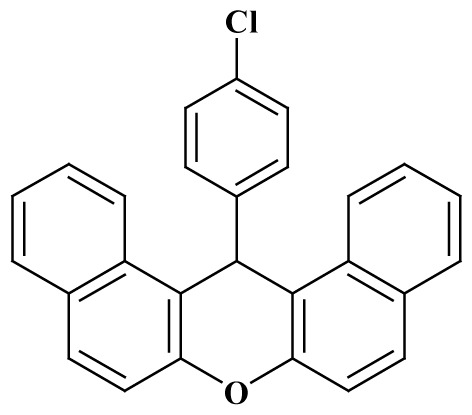	2	90	288–290	287–289 [33]
**3-iv**	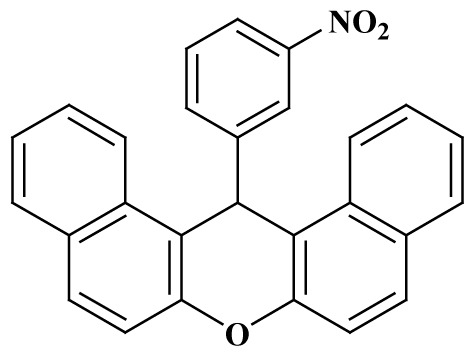	2	89	214–216	214–217 [34]
**3-e**	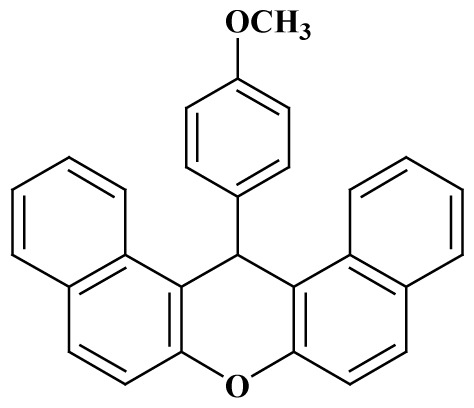	2.5	92	206–208	206–208 [35]
**3-f**	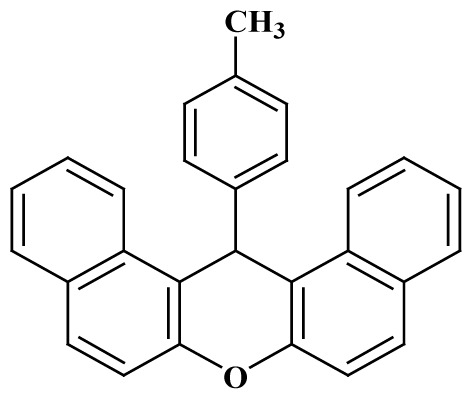	3	88	226–228	228–230 [35]

b (refers isolated yields.)

**Table 4:** Comparison of efficiency rates of the proposed mild acidic charcoal catalyst with some previously reported catalysts for the synthesis of dibenzoxanthenes.

Methods reported by	Catalyst used	Reaction conditions	Time	Yield (%)
Mirkhani et al. [36]	Carbon-based solid acid	DCM/reflux	9 h	94
Nagarapu et al. [37]	NaHSO_4_. SiO_2_	Solvent free/125 °C	8 h	88
Das et al. [38]	HClO_4_·SiO_2_	Solvent free/100 °C	3.5 h	92
Dabiri et al. [39]	Montmorillonite K10	Solvent free/100 °C	3 h	75
Tayebee and Tizabi [40]	H_5_PW_10_V_2_O_40_	Solvent free/100 °C	1 h	67
Sarma and Baruah [32]	H_2_SO_4_	AcOH/80 °C	73 h	55
Shakibaei et al. [41]	Dowex-50W	Solvent free/100 °C	1.5 h	78
**This work**	**Mild acidic charcoal**	**EtOH/reflux**	**2.5 h**	**94**

## Footnotes

1 Reza K, Monireh N (2016). Synthesis of 1H-tetrazole derivatives from amine compounds in the presence of copper nanoparticles on charcoal (CU/C) as a heterogeneous catalyst – Presented at the Iranian Seminar of Organic Chemistry. Website https://sid.ir/paper/936306/en [accessed 25 January 2022].
